# DEEP: a general computational framework for predicting enhancers

**DOI:** 10.1093/nar/gku1058

**Published:** 2014-11-05

**Authors:** Dimitrios Kleftogiannis, Panos Kalnis, Vladimir B. Bajic

**Affiliations:** 1 Computer, Electrical and Mathematical Sciences and Engineering Division (CEMSE), King Abdullah University of Science and Technology (KAUST), Thuwal 23955-6900, Saudi Arabia; 2 Computational Bioscience Research Center (CBRC), Computer, Electrical and Mathematical Sciences and Engineering Division (CEMSE), King Abdullah University of Science and Technology (KAUST), Thuwal 23955-6900, Saudi Arabia

## Abstract

Transcription regulation in multicellular eukaryotes is orchestrated by a number of DNA functional elements located at gene regulatory regions. Some regulatory regions (e.g. enhancers) are located far away from the gene they affect. Identification of distal regulatory elements is a challenge for the bioinformatics research. Although existing methodologies increased the number of computationally predicted enhancers, performance inconsistency of computational models across different cell-lines, class imbalance within the learning sets and *ad hoc* rules for selecting enhancer candidates for supervised learning, are some key questions that require further examination. In this study we developed DEEP, a novel ensemble prediction framework. DEEP integrates three components with diverse characteristics that streamline the analysis of enhancer's properties in a great variety of cellular conditions. In our method we train many individual classification models that we combine to classify DNA regions as enhancers or non-enhancers. DEEP uses features derived from histone modification marks or attributes coming from sequence characteristics. Experimental results indicate that DEEP performs better than four state-of-the-art methods on the ENCODE data. We report the first computational enhancer prediction results on FANTOM5 data where DEEP achieves 90.2% accuracy and 90% geometric mean (GM) of specificity and sensitivity across 36 different tissues. We further present results derived using *in vivo*-derived enhancer data from VISTA database. DEEP-VISTA, when tested on an independent test set, achieved GM of 80.1% and accuracy of 89.64%. DEEP framework is publicly available at http://cbrc.kaust.edu.sa/deep/.

## INTRODUCTION

Transcription regulation in human genes is a complex process (1,2). Promoters are cis-regulatory regions, which serve as anchor points for recruiting multiprotein complexes required for transcription. Although these regions have been extensively studied, their underlying transcriptional mechanism is not yet fully understood (3). Recent advances in high-throughput experiments like the 3C technology indicate that interactions between proximal and distal regulatory elements orchestrate gene expression between different cell types. In contrast to proximal elements, distal elements are not located near to the genes whose activity they affect, and can be located 20 kb or further away, or even can be located at different chromosomes. In addition, their functional mechanism appears to be independent of the upstream/downstream location of the genes they target. The better-characterized distal regulatory elements in eukaryotes are enhancers, silencers and insulators (4,5). Providing an accurate definition for these regulatory elements is not an easy task since they may have different roles depending on the cellular state (i.e. can be active or inactive, or can assume non-enhancer function) and their functional mechanism is not yet fully known. In the line with (6) we characterize enhancers as *cis*-acting DNA regulatory elements that increase the transcriptional output of the distal target genes. Enhancers activate gene transcription by recruiting transcription factors (TFs) and their complexes. For this reason, enhancer regions frequently contain clusters of binding sites of various TFs that vary across different cells and tissues. On the other hand, silencers, repressors and insulators have practically negative effects on the cellular transcriptional output either through recruitment of transcriptional repressor proteins (7), or by preventing the spread of heterochromatin (8).

Recent experimental procedures shed light on distal regulatory element interactions and decipher parts of their underlying operational mechanism. For instance, chromatin immunoprecipitation followed by massive sequencing (ChIP-Seq) determine the chromatin accessibility in different organisms, tissues and under different conditions. On the other hand, Cap Analysis of Gene Expression (CAGE), estimates the quantity of 5′ ends of messenger RNA in a cell. Projects, such as the ENCODE (9) and the NIH Epigenome Roadmap (10), released libraries of histone modification marks in human genome, whereas the FANTOM5 project (11) released CAGE-based transcription start sites (TSSs) in different cell types and tissues and enabled for the comprehensive identification of functional regulatory elements. Data that harbors the relevant information and the complexity of this information itself make identification of enhancers a challenging bioinformatics problem (12). Thus, the development of efficient computational models for predicting distal regulatory elements based on the recent high-throughput data emerges as a necessary requirement for understanding gene control mechanisms.

The first category of computational approaches for enhancer identification has been based on evolutionary conservation (13). However, recent results indicate that human regulatory elements show low conservation among different species and consequently they cannot be effectively characterized based on orthologous regions in other mammals (14). The second category is based on more sophisticated algorithms (15) that associate enhancers and promoters with certain types of histone modification marks and transcription regulators. However, since different types of histone modifications and regulators characterize enhancers, the developed models are not general enough. Other methodologies (16) use DNase I Hypersensitivity sites (DHSs) extracted from DNase-Seq data to characterize accessible DNA regions in various human cell types. Usage of DHSs combined with TF binding site (TFBS) motifs extracted from well-known databases (Transfac (17), Jaspar (18), HOCOMOCO (19)) is promising, but has several limitations. Lack of specificity for predicting different elements (enhancers or silencers or promoters) is considered the main drawback. In addition, strong dependency on sequence motifs may be problematic, since simultaneous binding of protein complexes to combinations of different motifs cannot be easily captured (20). The third category of developed methods utilizes machine learning (ML) algorithms and histone modification marks derived data, to increase the pool of candidate enhancers. ChromHMM (21) utilizes hidden Markov models (HMM) and unsupervised clustering of profiles in nine cell lines, while Segway (22) offers an alternative genomic segmentation based on a Dynamic Bayesian Network. CSI-ANN (23) introduces an Artificial Neural Network approach, while ChromaGenSVM (24) applies a support vector machines (SVM) classifier combined with a genetic algorithm for optimizing several steps in the recognition process. A Random Forest-based approach (RFECS) that studies multiple cell lines (25) has also been proposed. Recently, an SVM-based approach, EnhancerFinder (26), is proposed that integrates various types of data to improve enhancer prediction as compared to methods utilizing data from a single source. Finally, a forth category of developed methodologies utilizes FANTOM5 CAGE tags combined with other high-throughput data to detect active *in vivo*-transcribed enhancers across multiple tissues (27).

Although the ML-based methodologies increased the pool of predicted enhancers in various ENCODE cell lines, some key questions require further examination. These include lack of systematic analysis in enhancer's usage, performance inconsistency of computational models across different cell lines, class imbalance within the learning sets required for development of enhancer prediction models, limited number of training samples, data availability, strong dependencies on *ad hoc* rules from chromatin signatures and dominant dependencies on p300 binding sites and DHSs. Some of the above-mentioned problems are tackled by RFECS method. However, RFECS, as well as CSI-ANN and ChromGenSVM, rely strongly on p300 binding sites and/or DHSs for selecting positive training examples. Due to the above-mentioned reasons the existing computational models are often not general enough and sometimes show inconsistent performance across different data sets. One the other hand, EnhancerFinder (26) is trained on a small set of developmental enhancers data from the VISTA enhancer browser (28). The method shows improved performance and we believe that this can be attributed to a great extent to the use of variety of data types.

In this study we developed DEEP, a general ML framework for predicting enhancers. The DEEP framework contains three components, DEEP-ENCODE, DEEP-FANTOM5 and DEEP-VISTA. The components of DEEP are trained on data with diverse properties that describe enhancer's activity under different cellular conditions. From the technical point of view, DEEP utilizes a two-phase algorithm that reformulates the prediction problem into a binary classification task of chromosomal regions as being enhancer candidates or not. The first phase of DEEP uses an ensemble of SVMs, where many SVM models are trained using different subsets of the original data. In the second phase, decisions are aggregated and a simple ANN is used for deriving the final prediction. DEEP-ENCODE selects (from a big pool of available attributes) histone marks (ChIP-Seq) derived features from multiple ENCODE cell lines, DEEP-FANTOM5 uses tissue-specific sequence characteristics derived from FANTOM5 experimental data, while DEEP-VISTA uses sequence characteristics derived from developmental enhancer data achieved in VISTA database. Experimental results across different cell lines/tissues and comparison analysis with state-of-the-art methods convincingly demonstrate that DEEP is a general and robust framework for predicting enhancers, and can be used to complement other methods in enhancer prediction tasks.

## MATERIALS AND METHODS

### The DEEP-ENCODE model

DEEP framework builds two models derived from different data sources that relate to different experimental conditions and characterize diverse regulatory functions. The DEEP-ENCODE model specializes to predict enhancers from data coming from the ENCODE repository (http://genome.ucsc.edu/ENCODE/dataMatrix/encodeChipMatrixHuman.html) from where we constructed the training and testing sets. For the training sets we used Gm12878, Hep, H1-hesc and Huvec cell lines data. For testing the performance of the developed models and for exploring the generalization capabilities in a genome-wide manner, we used data from Hela and K562 cell lines. All the above-mentioned data sets are well studied and annotation maps for them also exist (29). The construction of the enhancer set (positive set) was based on the ENCODE integrative genomic annotation (30). This annotation utilizes unsupervised clustering techniques, as well as experimental data (TFs like CTCF or Pol2, DNase data and FAIRE arrays) to label non-overlapping genomic segments according to their functionality described by a total of 25 states. From this annotation we chose for training the set of most confident regions characterized as strong enhancers (enh). On the other hand, the non-enhancers (negative) data set contains random genomic loci (10 × the number of enhancer bins) not annotated as promoters or enhancers. Since there is no ‘gold standard’ of experimentally verified enhancers across variety of cellular conditions, cell types and tissues, we used as the reference the ENCODE annotation proposed by Hoffman *et al.* (30) as it is widely accepted by the research community and complements recent findings presented by Andersson *et al.* in (27). We kept a ratio 1:10 between positive and negative samples/bins and the data generation process followed the procedure proposed by CSI-ANN model (23). However, the original CSI-ANN model was trained using only 394 positive data samples from (31,32), while we used all strong enhancers from the training cell lines. For the construction of DEEP-ENCODE model we performed experiments with different sets of attributes including 11 histones and 351 sequence characteristics (described in the next section and summarized in Supplementary Table S4). We found that models trained using mixture of sequence and histone-derived attributes were not as effective as those obtained using only histone mark-derived characteristics. In addition, the small set of histone marks enabled for the application of a feature selection based on an exhaustive search that identified optimal set of attributes that differentiates considerably between different cell lines (Supplementary Table S5). Our final feature vector was compiled from ENCODE ChIP-Seq data containing the following 11 histone modification marks: H2AFZ, H3K27ac, H3K27me3, H3K36me3, H3K4me1 H3K4me2, H3K4me3, H3K79me2, H3K9ac, H3K9me3, H4K20me1. During data pre-processing we generated bins corresponding to 200 bp regions. Each row (histone mark) in a feature vector was scaled using min–max normalization to the [0,1] interval. This normalization technique does not affect the scaling of the testing data since it is applied independently to each cell line. Thus, the quality of the results is unbiased. Note that the results obtained from our experimentation with histone marks are in agreement with recent findings, which manifest that, the chromatin states that describe enhancers present cell-specific properties that vary across different cell lines (33). The DEEP-ENCODE model trained with sequence characteristics is also available (although it has lower performance) and it has advantages for new cell lines where histone modification mark data are not provided.

### The DEEP-FANTOM5 model

The DEEP-FANTOM5 model was implemented to predict enhancers that are specifically expressed in various organs and tissues. We used ‘genuine’ enhancers recently published by the FANTOM5 consortium (27). The data is publicly available at http://enhancer.binf.ku.dk/Pre-defined_tracks.html. For training models we chose without loss of generality enhancers coming from five vital organs: heart, brain, liver, lung and kidney. For testing the performance of the developed model we made predictions to all the other available FANTOM5 tissues. The negative set (non-enhancers) contains random genomic regions with the same minimum, maximum and mean length of the previous tissue-specific enhancers (10 × the number of enhancers) not included in any other list of enhancers published by the FANTOM5 consortium. For describing enhancers, we used 351 attributes derived from the sequences themselves. These include frequencies of 4 mono-nucleotides, 16 di-nucleotides, 64 tri-nucleotides, 256 tetra-nucleotides, as well as information on GpC islands, 2 aggregate frequencies for C+G, A+T, sequence length, number of bp and other 6 attributes coming from suitable combinations of the above-mentioned characteristics. The detailed description of the feature vector is provided in the Supplementary Materials. It is worth noting that in this model we did not apply any normalization procedure in the training and testing processes. For the construction of DEEP-FANTOM5 model we did not include histone marks information. The reason is that such data for the organs and tissues we studied is not available.

### The DEEP-VISTA model

The DEEP-VISTA model was trained on human *in vivo*-derived developmental enhancers that present extreme evolutionary conservation with mouse. We used enhancer data archived in VISTA enhancer browser (28). Data sets are publicly available at http://enhancer.lbl.gov/frnt_page_n.shtml. For training SVM models we selected all 1729 human enhancers. The negative set (non-enhancers) contains random genomic regions with the same minimum, maximum and mean length of the selected human enhancer regions (10 × the number of enhancers) not included in any list of enhancers published in VISTA. Similarly to DEEP-FANTOM5 model, we used 351 attributes derived from the sequences themselves. We did not apply any normalization procedure in the training and testing processes. Again we note that we did not include histone mark information since such data for the developmental enhancers set is not available.

### Implementing DEEP

Ensemble techniques have been successfully applied for training classifiers with highly unbalanced classes (34). Typically, in the ensemble approaches the majority class is partitioned into several subsets such that each of them has approximately equal number of samples as the minority class. When dealing with millions of samples in the minority class, a well-know variant partitions the minority class as well into disjoint subsets such that each of them contains the same ratio between positive and negative samples. Our DEEP-FANTOM5 and DEEP-VISTA models follow the first approach and partitions the majority class (non-enhancers) into 10 disjoint subsets. In order to achieve faster training and to handle millions of positive and negative samples, the DEEP-ENCODE model follows the second variant and partitions both positive and negative training samples into 1000 disjoint subsets so that each learning subset contains positive and negative samples in the proportion 1:10. For data sampling and partitioning we used simple random sampling without replacement. After data partitioning, each of the learning subsets is used to develop an SVM model with Gaussian kernel function. The development of multiple classifiers covering different partitions of the original data provides a better approximation of the original data distribution (35). Predictions of individual SVM classifiers are combined through an ANN to generate a final prediction. The inputs to this ANN are confidence scores (confidence scores are defined as the proportion of positive votes versus all votes for models from each cell line) obtained in the first layer of DEEP from the four cell line-/tissue-specific ensemble models. For DEEP-ENCODE 4 confidence scores are aggregated, whereas for DEEP-FANTOM5 we collect 5 scores from the underlying tissue-specific models. In the case of DEEP-VISTA, since we do not use data from multiple tissues/cell lines, the confidence scores are the votes aggregated from an individual ensemble SVMs. For tuning the ANN topology and select the optimal number of neurons we applied 5-fold cross-validation to the union of the data we used for training. For DEEP-ENCODE we trained on the union of data derived from Gm12878, H1, Hep and Huvec, whereas for DEEP-FANTOM5 we utilized brain, heart, lung, liver and kidney tissue data. The DEEP-VISTA model is trained on the union of subsets used for training individual SVMs. The best-trained model in terms of classification performance was utilized further for taking final decisions for all the cell lines and tissues we tested. We also experimented with other simple decision-making mechanisms like the majority voting and the experimental results for this version can be found in Supplementary Materials.

For the DEEP-ENCODE component, an ensemble SVM classifier was constructed for four cell lines (Gm12878, H1-hesc, Huvec and Hep). In total we trained 4000 (4 × 1000) classifiers. To do that, we partitioned the data randomly and we selected 20% of the samples for training and for tuning the model-specific classification parameters, while the remaining 80% of samples was kept for evaluating the performance of each individual ensemble model. For the DEEP-FANTOM5 component we followed the same logic and we trained an ensemble model with 10 classifiers for each of the lung, brain, heart, kidney and liver tissues (in total we trained 5 × 10 = 50 classifiers). We chose 40% of the original data for training and tuning and 60% for testing. For the DEEP-VISTA component again we followed the same approach and we trained an ensemble model with 10 classifiers using 20% of the original data for training and tuning and 80% for testing. The ratio between training and testing sets for each model was experimentally tuned taking into account the run time required for training DEEP model. A more detailed description is provided in the Supplementary Materials. Each model derived from one of the training data partitions utilizes an SVM classifier with Gaussian kernel function. When dealing with data containing unbalanced classes, SVM tend to be biased toward the majority class (34), but since we used an ensemble approach in both components of DEEP, this problem is reduced.

**Figure 1.**
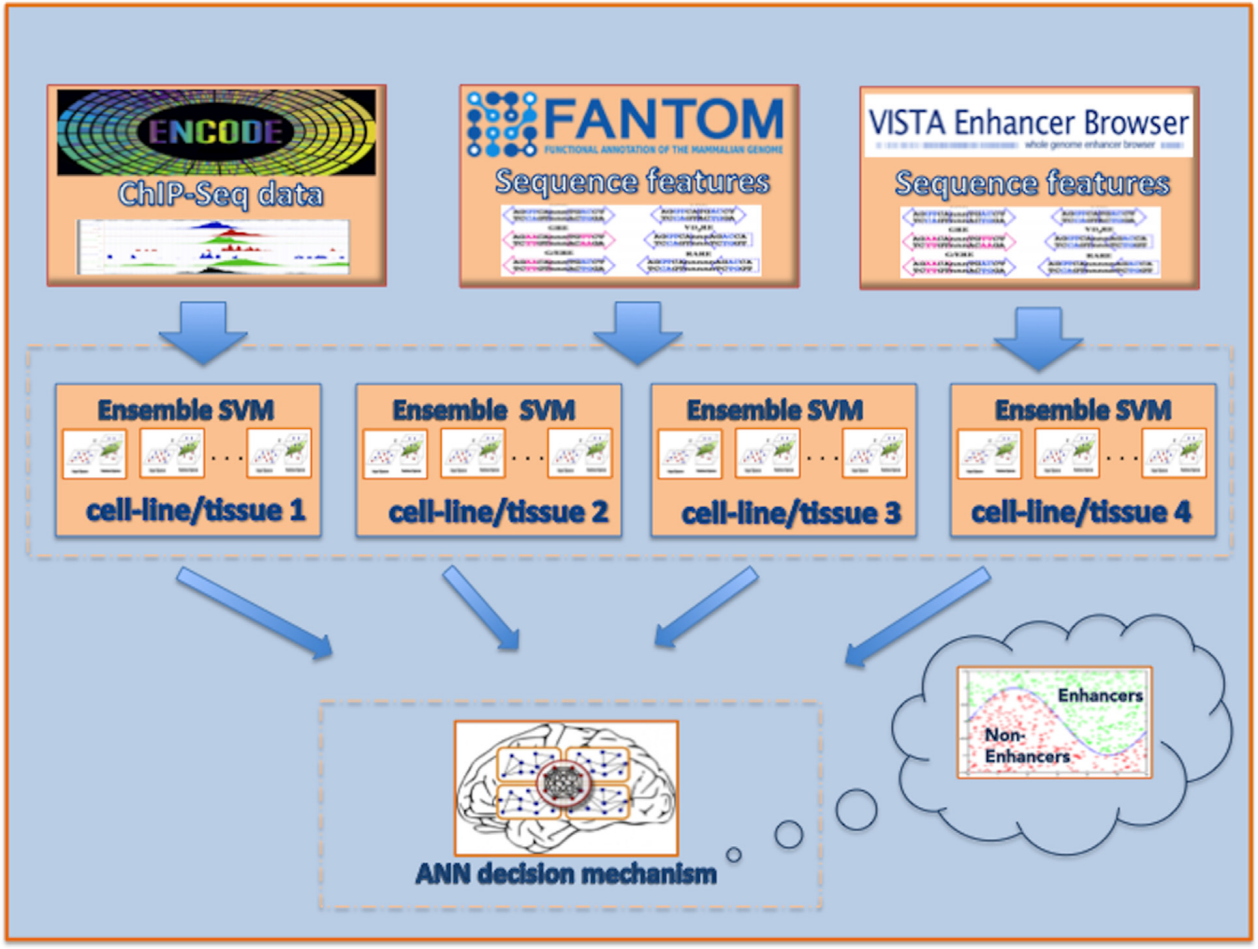
DEEP framework. The input to DEEP is either ChIP-Seq data for 11 histone marks or DNA sequences. After the feature vector computation, DEEP classifies the unknown instances using cell line-specific multiple SVM models or tissue-specific multiple SVM model. For each sample a confidence score is created based on the number of votes derived from each model. These confidence scores are passed to an ANN that takes the final decision and classifies each sample as candidate enhancer or not.

Tuning the SVM regularization parameter ‘C’ and the Gaussian kernel parameter ‘gamma’ was accomplished using a simple grid search algorithm (36). For every training round for simplicity and for saving time (note that we are training multiple SVM models) we selected randomly 70% of the training data for optimizing these two parameters. We computed all grid combinations of parameters and then we performed classification. We selected the case that maximizes the geometric mean (GM) of Specificity and Sensitivity. GM is a performance metric suitable for imbalanced data sets (37). In the second round of optimization, the same idea is applied to a fine-grained search space by increasing 10 times the step resolution. In the first step of the optimization technique the resolution of grid search was set to 0.2. Note that we applied logarithmic resolution in the range of (1,500) for parameter C and (0,50000) for parameter gamma, but any other resolution could also been applied. Figure 1 presents the DEEP workflow and describes DEEP utilization for classifying unknown data items.

A drawback we faced during the development of DEEP was the computational time required for training and tuning multiple individual SVM models. Similarly, the time required for predictions in unknown samples is significant because it requires classification over multiple individual SVM models. However, since the training data subsets and the models are totally independent, the implementation is fully parallelized. The most expensive part of our work was the training of DEEP-ENCODE component, because it requires training of 1000 individual models coming from each cell line (4000 in total). The computational time for training sequentially a cell-specific DEEP-ENCODE model is on average 12.8 h, which can be reduced to an average of 1.9 h in a workstation with 8 CPU cores and 196 GB RAM (Intel Xeon 2.6 GHz). Similarly, an optimized implementation for testing includes efficient partitioning of the data in chunks that fit into the main memory and can be fully parallelized as well.

The fact that we incorporated models derived from different ENCODE cell lines, various FANTOM5 tissues and developmental enhancers from VISTA into a unified framework for predicting enhancers increases the generalization ability and maximizes the capability of predicting enhancers in new cell lines, tissues and cellular conditions. The implementation of DEEP was made in Matlab R2012b and the standalone programs with the data sets used in this study are available at http://cbrc.kaust.edu.sa/deep/.

## RESULTS AND DISCUSSION

### Studying the performance of DEEP-ENCODE component

**Figure 2.**
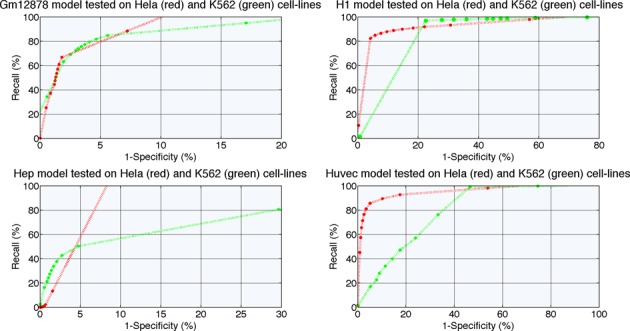
ROC performance curve for ensemble models trained on ENCODE cell line-specific data and tested on independent data coming from Hela and K562 cell lines.

To explore the effectiveness of individual models trained on information form one cell line to predict enhancers in other cell lines, we tested the performance of Gm12878, H1-hesc, Hep and Huvec ensemble classifiers on data from Hela and K562. The data normalization process required for the testing data has a limitation that it requires whole-genome ChIP-Seq signals in bigwig format for the unknown data items for testing (or an equivalent data format to convert). However, this is allowed because the ENCODE project releases whole-genome ChIP-Seq signals for the studied cell lines. Figure 2 present the ROC performance curve for these cell line-specific trained models for different decision thresholds.

A more thorough analysis of the generalization capabilities of individually deployed models revealed that few cell lines share a lot of the common properties and thus generalization becomes easier for such cases. In these situations, a prediction model derived for one cell line can be used to predict enhancers in such other cell lines. However, this is not a general property. For example, Gm12878 cell line has better predicting capabilities in K562 rather than in Hela. On the other hand, model derived from Huvec data generalizes well on data from Hela cell line, but achieves very poor performance on K562 data. Surprisingly, the H1 cell line-derived model generalizes well according to the ROC curve on data from both test cell lines, but more extensive analysis revealed very poor positive predictive value (PPV). The complete experimental setup that contains all the performance indicators accompanied with Precision–Recall curves is presented in Supplementary Material and is available at http://cbrc.kaust.edu.sa/deep. It becomes apparent that no model derived from single cell line data (of the type and within the framework we used) can effectively predict enhancers in all the other cell lines. That fact suggests a significant performance consistency challenge across various cell lines.

**Figure 3.**
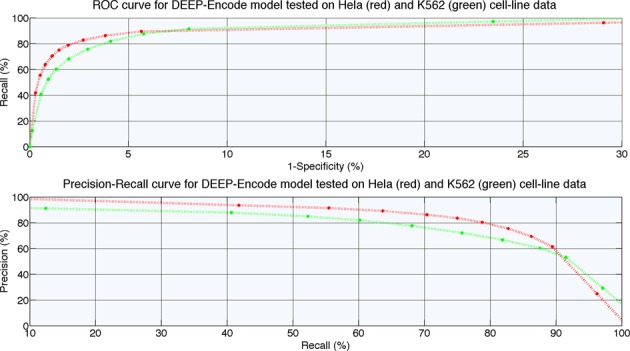
ROC and Precision–Recall performance curves for DEEP-ENCODE component tested on independent data coming from Hela and K562 cell lines.

The DEEP-ENCODE component resolves the above-mentioned issues and manifests greater generalization capabilities in both cell lines we used for testing. Figure 3 presents the ROC curve and the Precision and Recall performance curve. The decision-making schema we used, offers a threshold-free decision mechanism (threshold-free here refers to the thresholds that can be applied in steps 1 and 2 of the framework) by utilizing a simple ANN as the final output block of the DEEP-ENCODE component. The results illustrate that the combined two-layers framework with an ANN as the final decision maker generalizes better than individual models and achieves on average much better performance than other decision-making schemas that we evaluated. Comparing the results of DEEP-ENCODE with those obtained by the cell-specific models, we conclude that the combination of models achieves much better generalization capabilities. This advantage makes DEEP a robust tool for predicting enhancers in multiple cell lines.

### Performance comparison of DEEP-ENCODE with existing methods

To assess the capability of DEEP-ENCODE to predict effectively enhancers in a genome-wide manner, we used Hela and K562 cell line data and associated annotation from the ENCODE repository. These two cell line data were not used in the training process at all, so they are independent testing data for our evaluation. Here, in order to eliminate potential performance overestimation we eliminated enhancers that are common across the training sets of Gm12878, H1-hesc, Hep and Huvec models and the enhancer sets of Hela and K562 cell line data. Supplementary Table S12 presents the number of pairwise overlapped enhancer bases for all the data sets deployed in this study. To eliminate the overestimation of performance we excluded common bins between any of the Gm12878, H1, Hep and Huvec, training sets and the Hela and K562 testing sets. We also excluded from all training data sets, the enhancer regions that are described by exactly the same feature vectors as the enhancer regions in the test sets. After that filtering, we obtained 23 666 553 bp (0.764% of the genome) of enhancer predictions for Hela and 28 238 758 bp of enhancer predictions (0.912% of the genome) for K562. Since there is no baseline set of experimentally verified enhancers for these ENCODE cell lines, in order to have a fair comparison with respect to other methods, we evaluated predictions of enhancers through their overlap with experimental data that includes p300 ChIP-Seq peaks, DHS markers support (23–25) as well as RNA Polymerase II (Pol II) and TATA-binding protein (TBP) ChIP-Seq peaks. We computed different performance indicators. To provide a clear definition for them, first, we define the following sets:

- = the number of predicted enhancer bases that have p300/DHS experimental support
- = the total number of predicted enhancer bases
- = the total number of bases for p300/DHS experimental data
- = the number of predicted enhancer bases that overlap with promoters

Using the above annotation we determine the following performance indicators:

- Positive Predictive Value (PPV) = A/B
- Jaccard Index = A/(B + C − A)
- F1-score = 2*A/(B + C)
- Promoter overlap fraction (POF) = D/B

There are certain problems with the POF indicator since it is not straightforward to identify the promoter. The only real difference between the enhancers and promoters is the distance from the target genes, but in most cases we do not know the targets of enhancers. One should note that an enhancer that has remote target genes can contain a promoter of a proximal non-protein coding RNA (eRNA) genes (38), thus making it impossible in such cases to distinguish between the two. In fact, a large fraction of Pol II targets transcription in enhancers (38) resulting in this overlap. In addition, DNA regions characterized as promoters or enhancers in one phenotype could change in another phenotype (33) as chromatin states change. Further, there is no clear definition of the promoter boundaries. The upstream boundary of a promoter could be from 400 bp up to 15 000 bp upstream of TSS as used across different studies. To complicate the problem even more, there is no unique TSS for a gene (11), so it is difficult to define promoter relative to a gene loci. Due to all above-mentioned reasons, we measured the overlap through ChIP-Seq data for Pol II and TBP. All human protein-coding genes and many non-coding RNA genes are transcribed via Pol II, which positions over TSS. TBP binds to TATA-box and it is found in ∼24% of human genes in their core promoters (39). Therefore, we combined the presence of both Pol II ChIP-peak signals with TBP ChIP-peak signals to have a stronger evidence of promoter type regions. Next, we mapped the candidate promoter regions we found to the predictions obtained by the studied programs. However, the results should be considered with caution as the POF indicator defined using Pol II and TBP data has above-mentioned weaknesses. Note that in Supplementary Table S21 we present the additional results that measure overlap with candidate promoter regions defined based on Pol II ChIP-Seq peaks and well-annotated TSS set of RefSeq genes (accessed at 29 September 2014).

Note that, for the Hela cell line we used ENCODE p300 ChIP-Seq peaks (set C for p300) covering 8 199 111 bp, DHS markers (set C for DHS) covering 38 580 135 bp and 4 078 010 bp belonging to Pol2 and TBP ChIP-Seq peaks. Regarding the K562 cell line, 987 378 856 bp belong to p300 ChIP-Seq peaks (set C for p300), 43 893 777 bp belong to DHS markers (set C for DHS) and 3 747 145 bp belong to Pol2 and TBP ChIP-Seq peaks.

Next, we compared our predictions with those generated by four state-of-the-art predictors, namely, CSI-ANN, RFECS, ChromHMM and Segway on the same cell lines (Hela, K562) that represent independent test data for our method. For CSI-ANN, all predictions were obtained based on the optimal model proposed by the authors, trained on CD4+T cell line data (32). For RFECS the best model was based on the optimal subset of histone marks derived from H1-hesc cell line data. Predictions of Segway and ChromHMM can be found at http://www.broadinstitute.org/∼jernst/ROUND8_ChromHMM/. However, the way these ML-based methods generated their training sets (except for ChromHMM and Segway that use unsupervised learning) does not guarantee that there is no overlap of ‘genuine’ enhancer regions between their deployed training sets and the Hela and K562 testing sets used in our study. We are aware of this potential overestimation of performance for these two methods.

For the Hela cell line, CSI-ANN made 26 721 354 bp enhancer predictions covering 0.863% of the genome; RFECS predictions covered 87 487 722 bp (2.826% of the genome size), ChromHMM predicted 71 098 730 bp (2.296%) and Segway 125 256 834 bp (4.046%). For K562 cell line, CSI-ANN predicted 34 635 309 bp (1.118% of the genome size), RFECS predicted 130 723 329 bp (4.222%), ChromHMM predicted 111 659 937 bp (3.606%) and Segway predicted 283 814 425 bp (9.168%). The comparison analysis is summarized in Tables 1–7.

**Table 1.** PPV with p300/DHS data for Hela cell line

Program	E = number of predicted enhancer bases	E overlapped with p300 peaks	PPV based on the overlap with p300 peaks (%)	E overlapped with DHS peaks	PPV based on the overlap with DHS peaks (%)
DEEP-ENCODE	23 666 553	1 925 158	8.13	11 795 822	49.84
CSI-ANN	26 721 354	1 475 323	5.52	12 088 783	45.24
RFECS	87 487 722	5 241 662	5.99	18 149 583	20.74
ChromHMM	71 098 730	5 697 282	8.01	19 195 950	26.99
Segway	125 256 834	7 345 767	5.86	26 772 699	21.37

**Table 2.** PPV with p300/DHS data for K562 cell line

Program	E = number of predicted enhancer bases	E overlapped with p300 peaks	PPV based on the overlap with p300 peaks (%)	E overlapped with DHS peaks	PPV based on the overlap with DHS peaks (%)
DEEP-ENCODE	28 238 758	22 884 991	81.04	14 743 218	52.20
CSI-ANN	34 635 309	29 524 533	85.24	17 977 798	51.90
RFECS	130 723 329	92 392 750	70.67	19 082 602	14.59
ChromHMM	111 659 937	77 861 594	69.73	20 037 473	17.94
Segway	283 814 425	181 013 092	63.77	29 728 154	10.47

**Table 3.** Jaccard Index with p300/DHS data for Hela cell line

Program	Jaccard Index based on the overlap with p300 peaks	Jaccard Index based on the overlap with DHS peaks
DEEP-ENCODE	0.064	0.233
CSI-ANN	0.041	0.226
RFECS	0.055	0.161
ChromHMM	0.077	0.212
Segway	0.058	0.195

**Table 4.** Jaccard Index with p300/DHS data for K562 cell line

Program	Jaccard Index based on the overlap with p300 peaks	Jaccard Index based on the overlap with DHS peaks
DEEP-ENCODE	0.023	0.256
CSI-ANN	0.029	0.296
RFECS	0.090	0.122
ChromHMM	0.076	0.147
Segway	0.166	0.099

**Table 5.** F1-score for genome-wide predictions in ENCODE Hela cell line

Program	F1-score based on the overlap with p300 peaks (%)	F1-score based on the overlap with DHS peaks (%)
DEEP-ENCODE	12.12	37.90
CSI-ANN	7.99	37.02
RFECS	10.87	28.49
ChromHMM	14.36	35.00
Segway	11.00	32.68

**Table 6.** F1-score for genome-wide predictions in ENCODE K562 cell line

Program	F1-score based on the overlap with p300 peaks (%)	F1-score based on the overlap with DHS peaks (%)
DEEP-ENCODE	4.51	40.88
CSI-ANN	5.78	45.78
RFECS	16.52	21.84
ChromHMM	14.17	25.76
Segway	28.48	18.14

**Table 7.** Promoter overlap fraction in actual number of bases. In the parenthesis we report% fraction

Program	Percentage of predicted enhancer bases with Pol II+TBP regions in Hela	Percentage of predicted enhancer bases with Pol II+TBP regions in K562
DEEP-ENCODE	1 934 940 (8.17%)	1 831 793 (6.84%)
CSI-ANN	3 047 118 (11.40%)	2 785 755 (8.04%)
RFECS	620 220 (0.70%)	299 129 (0.22%)
ChromHMM	430 345 (0.60%)	160 847 (0.14%)
Segway	1 579 545 (1.26%)	1 330 238 (0.46%)

The comparison of the performances revealed that DEEP-ENCODE covers the smallest portion of the genome for both test cell lines followed by CSI-ANN, ChromHMM, RFECS and Segway. Based on PPV, DEEP-ENCODE always performs better than all the other methods relative to p300 and DHS support in both evaluated cell lines. Based on Jaccard Index, DEEP-ENCODE and ChromHMM share the best results followed by CSI-ANN, Segway and RFECS. Based on F1-score, DEEP-ENCODE and ChromHMM again are ranked first followed by CSI-ANN, RFECS and Segway in both evaluated cell lines. Finally, the smallest POF is achieved by ChromHMM method followed by RFECS and Segway.

Since, using different performance indicators the studied programs present advantages and disadvantages we rank their performance according to the four metrics described earlier. In total, 14 different tests were made (including 5 methods, 2 cell lines and 4 performance indicators). Following the ideas of (40) we averaged the ranked position of each of the five methods used in comparison in all of the 14 tests. Table 8 shows the overall score and average rank position for each of the methods. The lower the average rank position the better is the method. The analysis revealed that across the different performance tests DEEP-ENCODE is ranked first, followed by ChromHMM, CSI-ANN, Segway and RFECS. This fact convincingly demonstrates that DEEP-ENCODE performs well relative to the existing methods for enhancer predictions and can usefully complement them in this challenging task. Additional comparisons that measure the performance of the studied methods based on the same number of predictions (we used predefined thresholds and with random subsampling we selected the same amount of predicted enhancers) can be found in Supplementary Figures S3–S6. The performance curves for the overlap of predicted enhancer bases (obtained by the studied programs) with the ‘gold-standard enhancers’ obtained from (30) in a genome-wide manner can be found in Supplementary Figures S7 and S8.

**Table 8.** Relative ranking of ML methods that took part in the comparison study

	DEEP-ENCODE	CSI-ANN	RFECS	ChromHMM	Segway
PPV p300 (Hela)	1	5	3	2	4
PPV p300 (K562)	1	2	3	4	5
PPV DHS					
(Hela)	1	2	5	3	4
PPV DHS (K562)	1	2	4	3	5
Jaccard Index p300 (Hela)	2	5	4	1	3
Jaccard Index p300 (K562)	5	4	2	3	1
Jaccard Index DHS (Hela)	1	2	5	3	4
Jaccard Index DHS (K562)	2	1	4	3	5
F1-score p300 (Hela)	2	5	4	1	3
F1-score p300 (K562)	5	4	4	3	5
F1-score					
DHS (Hela)	1	2	5	3	4
F1-score					
DHS (K562)	2	1	4	3	5
POF (Hela)	4	5	2	1	3
POF (K562)	4	5	2	1	5
OVERALL RANKING	1st (32), 2.2	3rd (45), 3.2	5th (51), 3.6	2nd (34), 2.4	5th (56), 4

All p300 data, TBP data, DHS markers, candidate promoter regions, predictions obtained by the different programs, as well as scripts for reproducing the results are provided at http://cbrc.kaust.edu.sa/deep/.

### Validating DEEP-ENCODE genome-wide predictions using enhancer-related TF binding models

**Figure 4.**
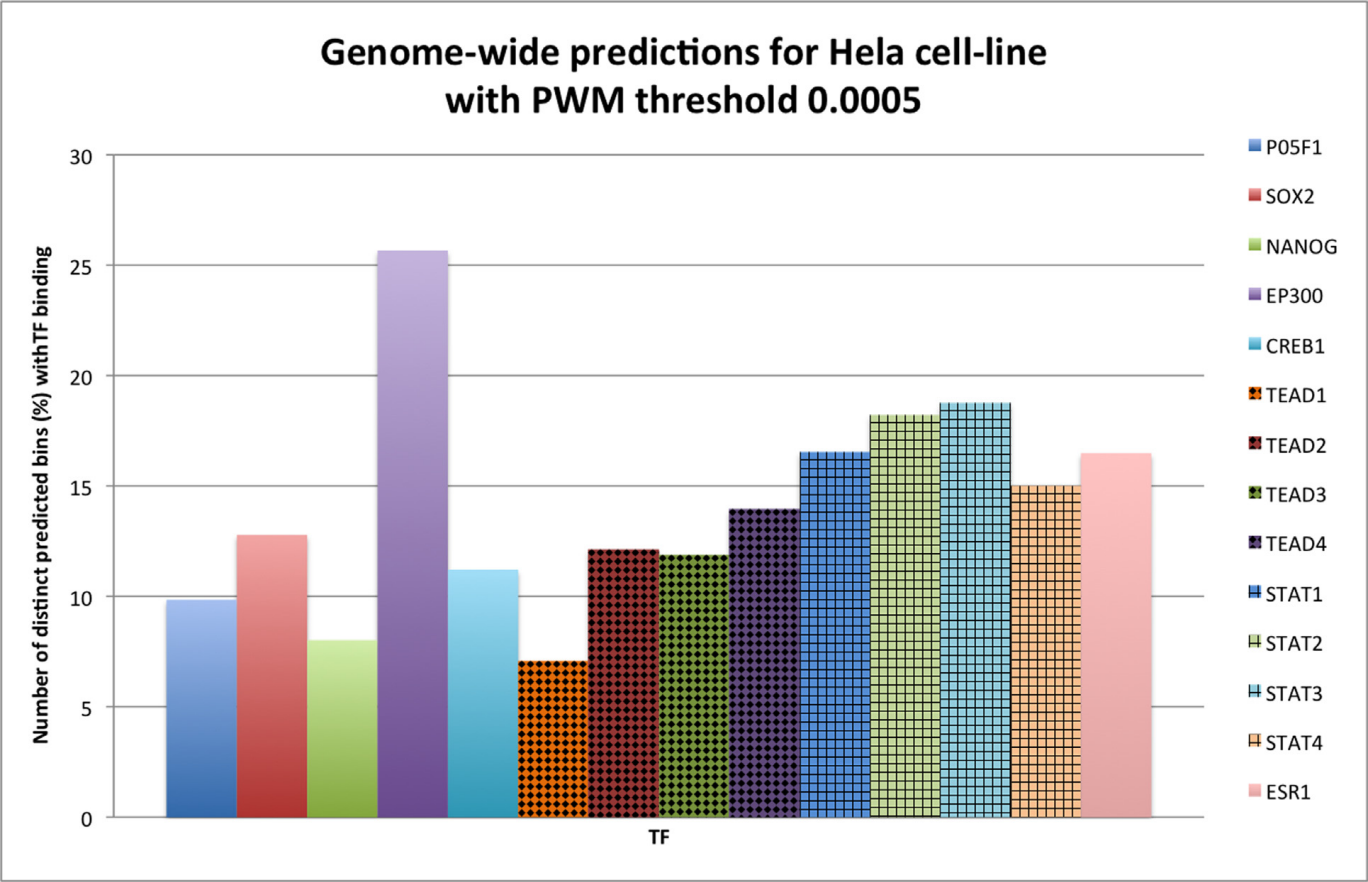
Number (%) of enhancer predictions (in bins) for Hela cell line that have at least one TFBS divided by the total number of enhancer predictions (PWM with threshold 0.0005).

**Figure 5.**
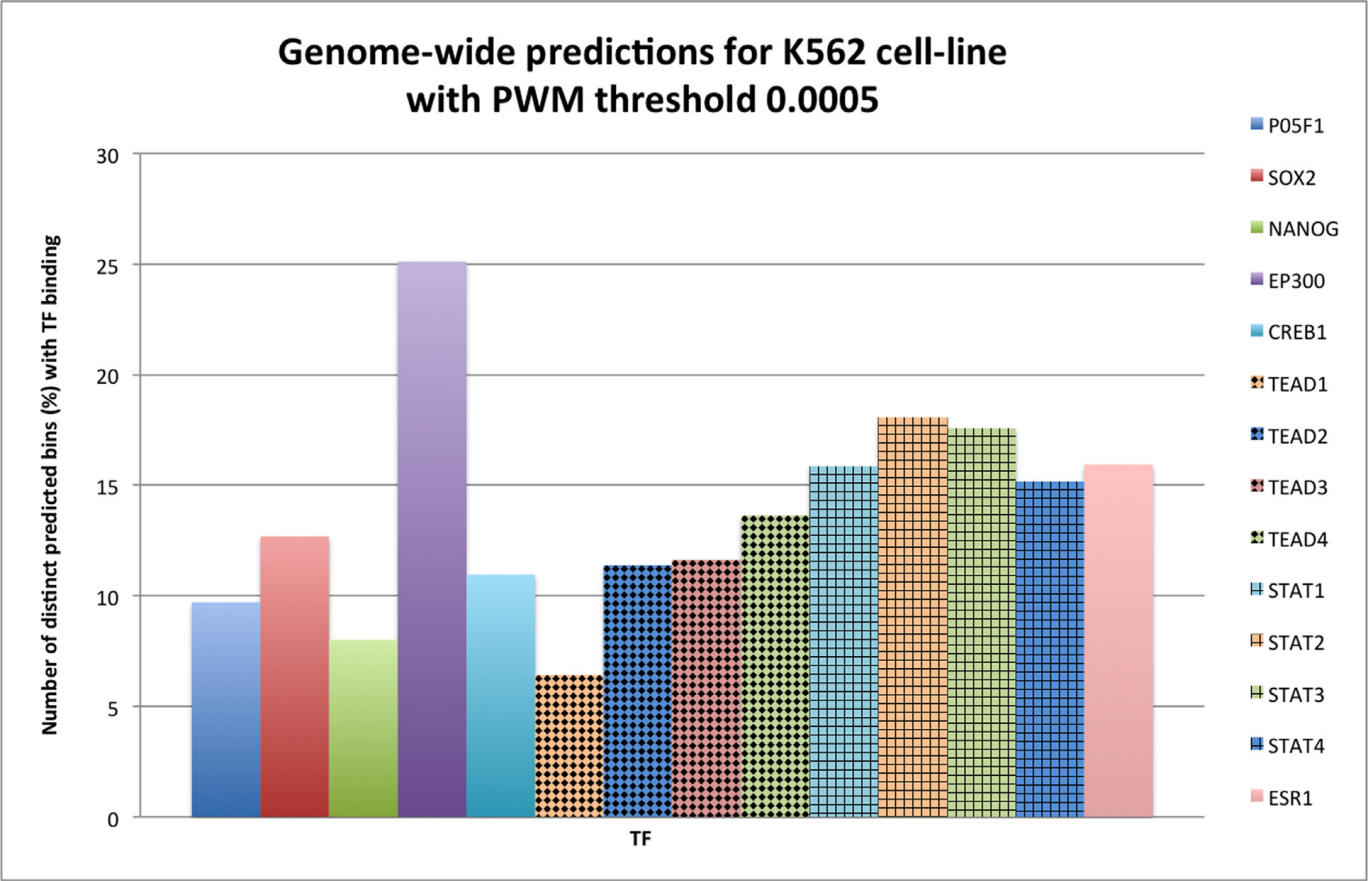
Number (%) of enhancer predictions (in bins) for K562 cell line that have at least one TFBS divided by the total number of enhancer predictions (PWM with threshold 0.0005).

Another indirect way of validating predicted enhancers involves enrichment of specific TFs described by positional weight matrixes (PWM) that bind to enhancer-predicted regions. Here, we tested binding of several TFs to genome-wide predictions obtained by DEEP-ENCODE model for Hela and K562 cell lines. We utilized HOCOMOCO database, which contains PWM models for 476 distinct TFs. From them, we selected a small subset that contains well known enhancer-related TFs like Oct2 (PO5F1), Sox2, Nanog, p300 (EP300), CBP (Creb1), TEAD1, TEAD2, TEAD3, TEAD4 (TEAD family), STA1, STAT2, STAT3, STAT4 (STAT family), TRAP220 (ESR1). Next, we mapped the enhancer sequences (and their reverse complements) against the subset of TFs using MOODS software (41). Figures 4 and 5 present an overview of the results for Hela and K562 cell lines. We reported% proportion of enhancer predictions in bins that have at least one TF hit as obtained by the PWM models divided by the total number of predicted bins. These results were obtained using a *P*-value threshold for binding equal to 0.0005. Results for *P*-value equal to 0.005 as well as the detailed list of hits are available in our web repository http://cbrc.kaust.edu.sa/deep/. We found that predictions obtained for both cell lines are enriched with putative binding sites of all selected TFs which all have been found in more than 5% of cases, with p300, STAT family TFs and TRAP220 being most prominent and being present in at least 15% of the cases.

### The performance of DEEP-FANTOM5 component on all the available FANTOM5 tissues

**Figure 6.**
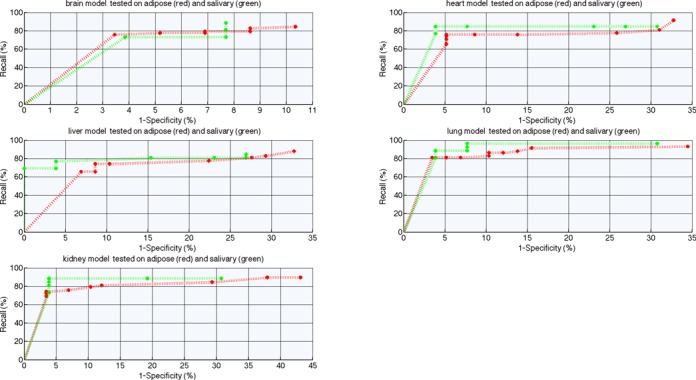
ROC performance curve for ensemble models trained on FANTOM5 tissue-specific data and tested on independent data coming from adipose and salivary.

**Figure 7.**
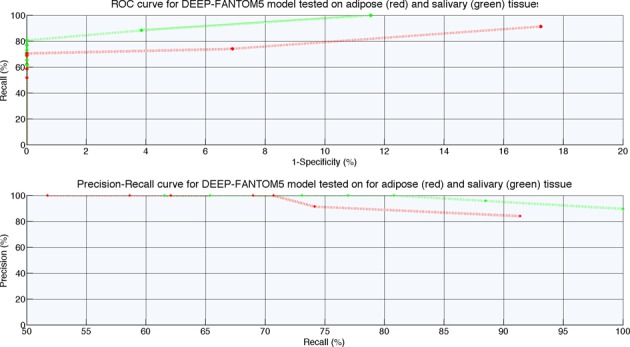
ROC and Precision–Recall performance curves for DEEP-FANTOM5 component tested on independent data coming from adipose and salivary.

Similarly to DEEP-ENCODE experiments, we explored the capacity of individual models trained in one tissue to predict enhancers in other tissues. To do so, we tested the performance of ensemble classifiers developed separately from brain, heart, lung, kidney and liver data, on data coming from adipose and salivary tissues. Figure 6 presents ROC performance curves for models trained on tissue-specific data for different decision thresholds. Note that the adipose and salivary tissues were chosen randomly for illustration purposes for Figures 6 and 7. Later, to assess the generalization capabilities of DEEP-FANTOM5 trained on data from a small subset of vital organs, we tested the performance on all the other available tissues from FANTOM5 repository and we present the detailed results in Supplementary Files. All the tested tissues are independent data sets and they did not take part in any training process deployed in DEEP. Moreover, in order to avoid potential overestimation of performance, we excluded enhancers regions of the tested tissues that overlap with enhancers from the training data. Supplementary Table S13 presents the number of enhancer regions before and after this filtering process. We applied strict filtering criteria meaning that we removed enhancer samples from the testing sets if they have at least 1 bp overlap with an enhancer regions used for training. It is worth noting that we did not apply any normalization procedure in the training and testing data. Then, we performed classification using the best models obtained for brain, heart, lung liver and kidney and we utilized the best-performing ANN model for making the final prediction. Figure 7 shows the ROC and Precision–Recall performance curves for DEEP-FANTOM5 model tested on adipose and salivary tissues.

For comparison purposes and in order to assess the predictive power of the developed models we used the maximum GM and accuracy that the models achieve and we computed the mean value across the 36 tested cases. DEEP-FANTOM5 is ranked first and achieves 90% (±0.047) GM and 90.2% (±0.046) accuracy. From the individual tissue-specific models the one trained on lung data is ranked second and achieves 89.9% GM (±0.067) and 90% (±0.064) accuracy followed by the model trained on kidney data that presents 89.1% (±0.061) GM and 89.3% (±0.059) accuracy on average. Although the measures of performance of GM and accuracy do not show much difference between the models derived from single tissue/organ data and DEEP-FANTOM5, we observe that DEEP-FANTOM5 models achieve almost always specificity and PPV of 1 accompanied with high sensitivity, which was not possible with the models derived from single tissue/organ data. Thus, the beneficial effect of models derived form multiple tissue/organs is that in many cases the number of false positive predictions will be significantly smaller with DEEP-FANTOM5. As compared to the ENCODE cell line-derived models, we observe that for FANTOM5 data models derived from single tissue/organ data are performing much better in predicting enhancers in other tissues. This fact somehow contradicts to the results we obtained with the ENCODE cell line-specific ensemble models which predict enhancers in other cell lines with much lower performance. In summary, DEEP-FANTOM5 extends predictions to a set of enhancers with much more diverse properties as compared to the ENCODE-derived enhancer data.

### Studying the performance of the DEEP-VISTA component

**Figure 8.**
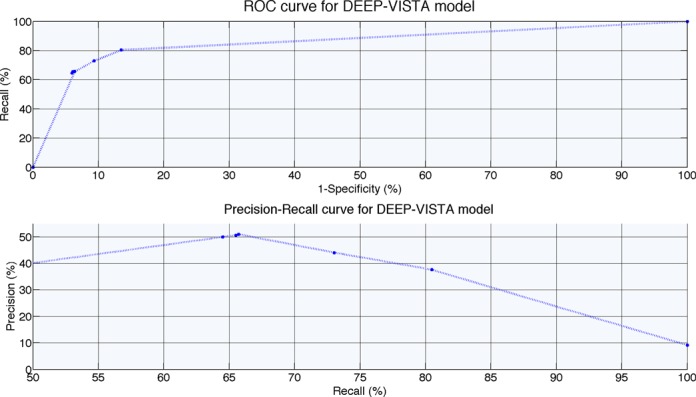
ROC and Precision–Recall performance curves for DEEP-VISTA component tested on independent data.

Similarly to the previous sections, here we explored the capability of DEEP-VISTA to predict developmental enhancers and discriminate them from other genomic regions. In the absence of multiple cell lines or tissues, we did not add multiple ensemble models in the first layer of DEEP-VISTA. In simpler words, DEEP-VISTA in its current implementation has only one ensemble SVM in its first layer (i.e. 10 individual SVMs) and the scores of these SVM models are aggregated through the second ANN layer to generate a prediction. Under this relation, we did not test the capability of DEEP-VISTA to predict enhancers in multiple tissues/cell lines as we did before. Figure 8 presents ROC performance curve and Precision–Recall curve. For this, we partitioned the original data to 20% for training and 80% for testing and we utilized the 20% for training and tuning SVM and ANN architectures. On the testing set DEEP-VISTA achieved GM of 80.1% and accuracy of 89.64%. Optimizing further the DEEP-VISTA component is an interesting task for the future when data from other cellular conditions become available.

## CONCLUSIONS

A novel computational framework involving three independent models is introduced for predicting enhancers based on ENCODE histone modification profiles and FANTOM5- or VISTA-derived sequence characteristics. To increase the generalization capabilities of the enhancer prediction models, we used, when available, either multiple cell lines/cell types or multiple tissues/organ as the training data, contrary to all previous enhancer predictors that used only single cell line/cell type data. The core component of the framework is the utilization of a two-layer ensemble classifier that trains multiple SVM cell line or tissue/organ models under the ensemble setting. The combination of different classification models under the ensemble setting provides greater generalization properties, reduces the class-imbalance problem, guarantees faster execution than training single models sequentially and achieves reliable performance across different data sets. Experimental results demonstrate that the DEEP framework applied on ChIP-Seq ENCODE data achieves higher performance than individual cell-specific ensemble models. Also, when it is applied for genome-wide predictions, it identifies enhancer candidates with higher precision than predictions obtained by four state-of-the-art programs. Moreover, DEEP integrates two additional components called DEEP-FANTOM5 and DEEP-VISTA, which streamline the analysis of enhancer's properties in multiple FANTOM5 tissues/organs and a specific set of developmental enhancers, respectively. DEEP-FANTOM5, when tested on identified enhancers regions from 36 different tissues achieves 90% GM and 90.2% accuracy on average. When tested on an independent test set, DEEP-VISTA achieved GM of 80.1% and accuracy of 89.64%. The incorporation of tissue-specific expressed enhancers in the DEEP framework shows that DEEP could have useful application in human genetics. Nonetheless, there is a room for further improvements. Adding more cell line-specific and tissue-specific models in the first layer of DEEP-ENCODE, DEEP-FANTOM5 or DEEP-VISTA components is anticipated to enhance further the generalization capabilities of DEEP. The performance of DEEP-FANTOM5 or DEEP-VISTA may be improved with an effective feature selection technique. Finally, the implementation of a web-based version of DEEP will also be an important task for the future development.

## SUPPLEMENTARY DATA

Supplementary Data are available at NAR Online.

SUPPLEMENTARY DATA
