# Supplementary material for: DEEP: a general computational framework for predicting enhancers
Source: Nucleic Acids Res. 2014 Nov 5;43(1):e6. doi: 10.1093/nar/gku1058 (PMC4288148; doi:10.1093/nar/gku1058)
Supplement: SUPPLEMENTARY DATA [file supp_43_1_e6__index.html]

DEEP: a general computational framework for predicting enhancers — DEEP: a general computational framework for predicting enhancers — SUPPLEMENTARY DATA 

# DEEP: a general computational framework for predicting enhancers

## SUPPLEMENTARY DATA

**Files in this Data Supplement:**

- SUPPLEMENTARY DATA
