# Supplementary material for: DEEP: a general computational framework for predicting enhancers
Source: Nucleic Acids Res. 2014 Nov 5;43(1):e6. doi: 10.1093/nar/gku1058 (PMC4288148; doi:10.1093/nar/gku1058)
Supplement: SUPPLEMENTARY DATA [file supp_gku1058_nar-01727-met-z-2014-File018.pdf]

DEEP: A general computational framework for predicting enhancers

Dimitrios Kleftogiannis[1], Panos Kalnis[1] and Vladimir B. Bajic[2*]

[1] Computer, Electrical and Mathematical Sciences and Engineering Division (CEMSE), King Abdullah University of Science and Technology (KAUST), Thuwal, 23955-6900, Saudi Arabia.
[2] Computational Bioscience Research Center (CBRC), Computer, Electrical and Mathematical Sciences and Engineering Division (CEMSE), King Abdullah University of Science and Technology (KAUST), Thuwal, 23955-6900,Saudi Arabia

SUPPLEMENTARY MATERIAL

Selection of cell-lines and tissues: Number of positive and negative samples

In ENCODE repository there are 6 cell-lines from tier 1 and tier 2 experiments that are characterized by the same 11 histone mark data (with the exception of H3K9me1). For the training of our model we chose randomly 2 cell-lines from tier 1 experiments and other 2 from tier 2. These 4 cell-lines are: Gm12878, H1-Hesc, Hep-G2 and Huvec. The other two cell-lines, K562 (tier 1) and Hela (tier 2), are used for testing. We did not extend our experiments to tier 3 data since these data are of much lower quality. For these 6 cell-lines annotation maps from Hoffman et al 2013 are available. The number of positive and negative bins is presented in Supplementary Table 1.

DEEP-FANTOM5 component implements tissue-specific models coming from different FANTOM5 tissues. Specifically, we chose 5 tissues from vital organs for training. All other were used for testing. Supplementary Table 2 presents the number of positive samples as well as characteristics related to these tissue-specific candidate enhancer regions. Note that for the number of negative samples we chose 10 times the number of positive regions and we created tissue-specific negative datasets. During the negative dataset generation we preserved the positive dataset distribution meaning that we constructed negative samples randomly with the same minimum, maximum and mean length as the positive enhancer regions.

Selecting relevant features

To build efficient models usually requires removing redundant features and may result in the selection of a small and optimized feature set. The small number of more relevant features can sometimes improve the prediction performance of the deployed models and result in faster, more reliable and more cost-effective classification. For the DEEP-ENCODE model, we initially tested the classification performance using three different feature subsets. One subset consisting of 11 histone marks, the other one that contains 351 sequence-derived attributes, and the third that contains all the available features (362 in total). However, when we used all features or when we used only the sequence-derived features we achieved much lower performance compared to the one obtained with feature set that contains only 11 histone marks. For this reason the DEEP-ENCODE model is trained using this small subset of features. In addition, the relative small number of features (only 11 attributes) allows for the application of an exhaustive feature selection procedure. The exhaustive feature selection measures the classification performance for all possible combinations of features, which in our case are 2048 combinations.

For each cell-line we chose randomly 20% of the original data (we preserved the ratio 1:10 between positive and negative classes) and we performed classification using the same algorithm as the one deployed in DEEP-ENCODE component. We chose the combination of features that maximizes the geometric mean (GM) of sensitivity and specificity.

Supplementary Table 5 presents the results. Note that the whole process of exhaustive search took around 7 days to finish (sequential implementation in a Intel Xeon 2.5 GHz workstation).

A closer look to the previous feature subsets shows that different sets of features appear optimal for different cell-lines. We also observe that attribute H3K4me1 is always selected. This is consistent with the experimental evidence that associate this histone mark with enhancers. Apart from that, H3K9ac is selected in 5 out of 6 cell-lines, whereas H3K4me2, H3K4me3 and H3K36me3 are selected in 4 out of 6 cell-lines.

For the DEEP-FANTOM5 component it was impossible to perform exhaustive search over 351 characteristics. Instead of that, we utilized a wrapper-based feature selection tool (http://www.cbrc.kaust.edu.sa/dwfs/ that utilizes a GA for identifying relevant features. We chose the default setting and we selected features using Naïve Bayes classifier since it is much faster than other classification techniques available in this tool. Similarly to the DEEP-ENCODE component, selecting features for different tissues results in different relevant feature subsets. However, the performance we obtained using these selected features was always lower than using the performance achieved using the original feature vector. For this reason, we decided to use the original feature vector containing 351 features for the rest of our experimentation. However, identifying a more compact feature subset for this specific component remains a challenging task to be done in future.

Feature vector description used for exhausting search
A description of the 11 histone marks used for exhaustive search of the best feature combination for DEEP-ENCODE is presented in Supplementary Table 3.

Feature vector for DEEP-FANTOM5 and DEEP-VISTA
The DEEP-FANTOM5 and DEEP-VISTA components use 351 features derived from the DNA sequence itself. Supplementary Table 4 describes the feature categories. Note that ChIP-seq histone modification data for the FANTOM5/VISTA tissues are not currently available.

Tuning the ratio between testing and training
Since we chose the simple holdout approach (2-fold cross validation) for assessing the classification performance of the cell-line/tissue specific models, one important question that arises is how many positive and negative samples are sufficient to use for training. To resolve this issue we utilized an internal trial-error approach. Specifically, we performed multiple trainings with different ratios starting from 10% for training and 90% for testing, and progressively decreasing the number of testing samples (and increasing the number of negative). We considered the geometric mean of specificity and sensitivity and the positive predictive value as the most indicative performance metrics for this task. Supplementary Tables 6-11 presents the classification results. For DEEP-ENCODE component we observe that selecting the 20% of the data for training and 80% of the data for testing is most suitable and also the training time is reduced. Note that we were training thousands of SVM models. Similarly, for DEEP-FANTOM5 component we observe that 40% of the data for the training and 60% of the data for testing appears to be a suitable choice.

Testing the effectiveness of other decision-making mechanisms
In the ensemble techniques, there are many ways to combine decisions from individual models and draw the final predictions. We experimented with the simple voting schema applied to the DEEP-ENCODE component as follows: First, we applied predefined thresholds for phases 1 and 2. We considered different proportions of voting for making a final decision and we reported results by applying various decision thresholds starting from 10% up 100% of total votes, meaning that we required at least 100, 200, 300, 400, 500, 600, 700, 800, 900, 950, 960, 970, 980, 990 and 1000 classifiers to vote for the positive class. Supplementary Figure 1 presents the ROC performance curve. It becomes apparent that the ANN based decision mechanism is more sophisticated and has significant advantages over this simple voting schema. Thus, we applied the ANN decision mechanism for the experimentation presented in the manuscript.

Individual ENCODE cell-line specific models achieve poor Positive Predictive Values (PPV)

In order to examine further the capabilities of models for predicting enhancers trained on single cell-lines, we generated Precision-Recall curves for several cell-lines and tissues. Supplementary figure 2 presents these results. Overall, we show that cell-specific models achieve inconsistent performance across different cell-lines and their precision is very low compared to DEEP-ENCODE component.

Classification performance of DEEP-ENCODE with different feature subsets

To measure the effectiveness of different feature subsets used for the DEEP-ENCODE model, we calculated the average classification performance achieved using sequence-derived features and feature set that contains all the 362 attributes. We also measured the performance using different ratios of positive and negative samples. Supplementary Tables 14,15,16,17,18,19 present these results.

TABLES AND FIGURE LEGENDS

Supplementary Table 1: Number of positive and negative training samples/bins per ENCODE cell-line

Cell-line	Number of negative bins	Number of positive bins
Gm12878	1,936,200	193,620
H1	809,800	80,980
Hep	1,397,700	138,770
Huvec	3,283,000	328,300

Supplementary Table 2: Number of positive and negative training samples for FANTOM5 tissues used for training.

Tissue	Number of positive samples	Number of negative samples	Min length	Max length	Mean length
Kidney	124	1,240	30	1367	327.24
Heart	295	2950	18	1594	387.05
Lung	217	2,170	48	1541	358.56
Liver	84	840	59	1471	371.32
Brain	639	6390	18	1594	394.76

Supplementary Table 3: Description of histone modification marks

Histone modification	Brief Description
H2AFZ	Variant of H2A
H3K27ac	Detects Acetylation
H3K27me3	Detects trimethylation of Lysine 27
H3K36me3	Marks actively transcribed regions
H3K4me1	Associated with enhancers
H3K4me2	Marks promoters and enhancers
H3K4me3	Associated with active promoters
H3K79me2	Marks transcriptional transition regions
H3K9ac	Marks promoters in chromatin regions
H3K9me3	Associated with silenced chromatin
H4K20me1	Associated with active and accessible regions

Supplementary Table 4: DEEP-FANTOM5 and DEEP-VISTA feature set description

Category	Number of features	Description
Di-nucleotide frequency	16	XY where X,Y ε {A,C,G,T}
Tri-nucleotide frequency	64	XYZ where X,Y,Z ε {A,C,G,T}
Tetra-nucleotide frequency	256	XYZK where X,Y,Z,K ε {A,C,G,T}
Single Base frequencies	4	X where X ε {A,C,G,T}
Aggregate frequencies	2	A+T, C+G
Base pairs	1	The number of base pairs in the sequence
Length of sequence	1	The actual length of the sequence

GpC islands	1	GC/(sum(C)*sum(G)*length)
Miscellaneous	6	1. |sum(C)-sum(G)|/base pairs<br>2. |sum(A)-sum(T)|/base pairs<br>3. sum(A)/sum(T)<br>4. sum(C)/sum(G)<br>5. (sum(G)*sum(C))/length<br>6. (sum(A)*sum(T))/length

Supplementary Table 5: ENCODE cell-line specific relevant feature subsets

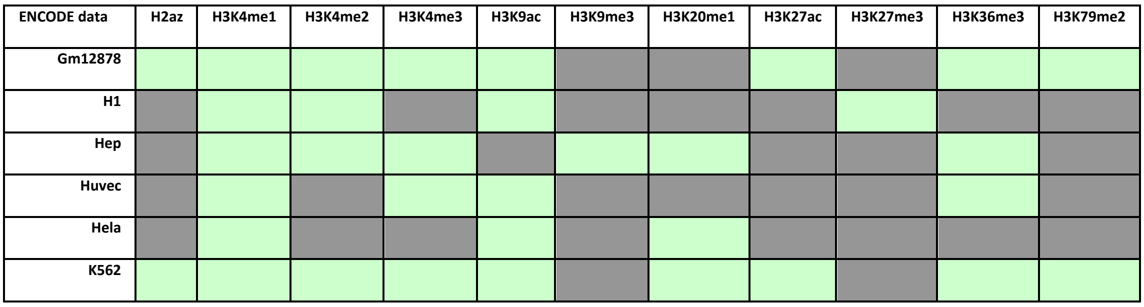

Supplementary Table 6: Performance of Gm12878 cell-line ensemble model using different portions of training and testing samples. In the first column we report % portion of training and % portion of testing samples.

**Gm12878**	**ACC**	**SEN**	**SPE**	**GM**	**PPV**	**NPV**
**10-90**	94.41	78.55	95.99	86.78	66.84	97.82
**20-80**	94.37	86.64	95.08	90.76	64.31	98.68
**30-70**	94.35	87.78	95.00	91.31	63.90	98.73
**40-60**	94.33	87.29	95.04	91.07	63.96	98.68
**50-50**	94.36	86.56	95.14	90.73	64.25	98.61

Supplementary Table 7: Performance of H1 cell-line ensemble model using different portions of training and testing samples. In the first column we report % portion of training and % portion of testing samples.

**H1**	**ACC**	**SEN**	**SPE**	**GM**	**PPV**	**NPV**
**10-90**	92.36	70.05	94.59	81.20	58.62	96.95
**20-80**	92.60	72.87	94.58	82.96	58.42	97.22
**30-70**	92.39	74.54	94.18	83.74	57.07	97.37
**40-60**	92.33	75.72	93.99	84.32	56.47	97.49
**50-50**	92.46	74.32	94.13	83.64	57.01	97.40

Supplementary Table 8: Performance of Hep cell-line ensemble model using different portions of training and testing samples. In the first column we report % portion of training and % portion of testing samples.

**Hep**	**ACC**	**SEN**	**SPE**	**GM**	**PPV**	**NPV**
**10-90**	94.28	75.20	96.18	84.98	67.09	97.49
**20-80**	94.42	79.11	95.95	87.09	66.65	97.87
**30-70**	94.41	81.13	95.73	88.11	65.92	98.07

**40-60**	94.32	82.69	95.49	88.83	65.02	98.22
**50-50**	94.31	83.65	95.37	89.29	64.69	98.32

Supplementary Table 9: Performance of heart tissue ensemble model using different portions of training and testing samples. In the first column we report % portion of training and % portion of testing samples.

Heart	ACC	SEN	SPE	GM	PPV	NPV
**10-90**	74.62	73.09	74.77	73.88	22.69	96.53
**20-80**	77.18	79.11	76.99	77.96	26.52	97.37
**30-70**	78.36	71.94	79.00	75.37	25.49	96.58
**40-60**	82.15	80.23	82.43	81.15	32.83	97.67
**50-50**	82.47	76.26	83.09	79.46	33.59	97.24

Supplementary Table 10: Performance of brain tissue ensemble model using different portions of training and testing samples. In the first column we report % portion of training and % portion of testing samples.

Brain	ACC	SEN	SPE	GM	PPV	NPV
**10-90**	80.30	79.17	80.41	79.66	30.56	97.49
**20-80**	82.75	82.92	82.73	82.70	34.86	97.99
**30-70**	80.99	85.50	80.54	82.91	31.86	98.24
**40-60**	85.28	83.21	85.49	84.25	38.34	98.08
**50-50**	85.72	79.72	86.32	82.85	39.41	97.71

Supplementary Table 11: Performance of liver tissue ensemble model using different portions of training and testing samples. In the first column we report % portion of training and % portion of testing samples.

Liver	ACC	SEN	SPE	GM	PPV	NPV
**10-90**	62.79	79.73	61.11	67.59	18.94	97.13
**20-80**	66.97	82.54	65.42	73.02	20.19	97.44
**30-70**	73.50	78.45	73.01	75.53	22.82	97.19
**40-60**	74.49	74	75.54	74.13	22.60	96.68
**50-50**	75.58	66.90	76.45	71.47	22.23	95.86

Supplementary Table 12: ‘Genuine‘ enhancer regions that are common across the DEEP-ENCODE training sets of different cell-lines. We report actual number of bases. In the parenthesis we report number of bins.

	Gm12878	H1	Hep	Huvec	Hela	K562
**Gm12878**		117,029 (584)	180,951 (904)	709,768 (3,548)	363,800 (1,819)	549,730 (2,748)
**H1**	117,029 (584)		100,574 (502)	347,190 (1,735)	164,418 (822)	206,446 (1,032)
**Hep**	180,951 (904)	100,574 (502)		543,860 (2,719)	435,754 (2,178)	335,601 (1,678)
**Huvec**	709,768 (3,548)	347,190 (1,735)	543,860 (2,719)		1,883,208 (9,416)	1,115,166 (5,575)
**Hela**	363,800 (1,819)	164,418 (822)	435,754 (2,178)	1,883,208 (9,416)		760,515 (3,802)
**K562**	549,730 (2,748)	206,446 (1,032)	335,601 (1,678)	1,115,166 (5,575)	760,515 (3,802)	

Supplementary Table 13: ‘Genuine‘ enhancer regions common across the DEEP-FANTOM5 training sets of different tissues. We report actual number of samples. The last column also

provided the accuracy of DEEP-FANTOM5 model tested on the original enhancer regions (without the filtering)

Tissue	Original enhancer regions	Enhancer regions non-overlapped with data used for training
adipose	108	58
Blood vessel	158	64
Esophagus	134	80
Female gonad	90	43
Gallbladder	81	48
Internal male genitalia	168	118
Large intestine	209	59
Lymph	30	17
Meninx	97	41
Olfactory region	11	1
Pancreas	35	6
Parotid	26	13
Pennis	21	11
Placenta	92	64
Prostate	115	69
Salivary	59	26
Skeletal muscle	95	35
Skin of body	20	6
Small intestine	143	86
Smooth muscle	66	23
Spinal cord	42	7
Spleen	277	169
Stomach	20	4
Submandibular	38	18
Testis	644	518
Throat	125	40
Thymus	347	278
Thyroid	142	72
Tongue	133	83
Tonsil	146	82
Umbical	10	6
Urinary bladder	105	61
Uterus	157	84
Vagina	62	32

Supplementary Table 14: Performance of H1 cell-line ensemble model using 351 sequence characteristics and different portions of training and testing samples. In the first column we report % portion of training and % portion of testing samples.

H1	ACC	SEN	SPE	GM	PPV	NPV
**10-90**	0.8736	0.0948	0.9515	0.2621	0.3164	0.9132
**20-80**	0.8621	0.1246	0.9359	0.3415	0.1627	0.9145
**30-70**	0.8704	0.1026	0.9472	0.2865	0.2569	0.9135
**40-60**	0.8684	0.1091	0.9443	0.2997	0.2269	0.9138
**50-50**	0.8650	0.1165	0.9399	0.3129	0.2007	0.9142

Supplementary Table 15: Performance of H1 cell-line ensemble model using all features (362) and different portions of training and testing samples. In the first column we report % portion of training and % portion of testing samples.

H1	ACC	SEN	SPE	GM	PPV	NPV
**10-90**	0.8904	0.0558	0.9739	0.1943	0.5039	0.9117
**20-80**	0.8909	0.0727	0.9727	0.2295	0.5001	0.9130
**30-70**	0.8944	0.0844	0.9754	0.2663	0.4960	0.9142
**40-60**	0.8971	0.1004	0.9768	0.2956	0.5135	0.9157
**50-50**	0.8972	0.1249	0.9744	0.3315	0.5110	0.9178

Supplementary Table 16: Performance of Hep cell-line ensemble model using 351 sequence characteristics and different portions of training and testing samples. In the first column we report % portion of training and % portion of testing samples.

Hep	ACC	SEN	SPE	GM	PPV	NPV
**10-90**	0.9066	0.0007	0.9972	0.0083	0.0633	0.9089
**20-80**	0.9070	0.0015	0.9975	0.0123	0.0733	0.9090
**30-70**	0.9038	0.0070	0.9934	0.0348	0.0813	0.9091
**40-60**	0.8955	0.0203	0.9830	0.0812	0.0879	0.9094
**50-50**	0.8771	0.0571	0.9591	0.2341	0.1225	0.9105

Supplementary Table 17: Performance of Hep cell-line ensemble model using all features (362) and different portions of training and testing samples. In the first column we report % portion of training and % portion of testing samples.

Hep	ACC	SEN	SPE	GM	PPV	NPV
**10-90**	0.9084	0.0002	0.9993	0.0058	0.1208	0.9090
**20-80**	0.9093	0.0095	0.9993	0.0427	0.2372	0.9098
**30-70**	0.8476	0.2524	0.9071	0.4785	0.2137	0.9239
**40-60**	0.8567	0.5035	0.8920	0.6702	0.3180	0.9473
50-50	0.8794	0.4388	0.9235	0.6366	0.3644	0.9427

Supplementary Table 18: Performance of Gm12878 cell-line ensemble model using 351 sequence characteristics and different portions of training and testing samples. In the first column we report % portion of training and % portion of testing samples.

Gm12878	ACC	SEN	SPE	GM	PPV	NPV
**10-90**	0.8354	0.2354	0.8954	0.4485	0.1846	0.9215
**20-80**	0.8274	0.3000	0.8801	0.5138	0.2001	0.9263
**30-70**	0.8272	0.2993	0.8800	0.5132	0.1997	0.9262
**40-60**	0.8276	0.2998	0.8804	0.5138	0.2004	0.9263
**50-50**	0.8268	0.3007	0.8795	0.5143	0.1997	0.9263

Supplementary Table 19: Performance of Gm12878 cell-line ensemble model using all features (362) and different portions of training and testing samples. In the first column we report % portion of training and % portion of testing samples.

Gm12878	ACC	SEN	SPE	GM	PPV	NPV
**10-90**	0.8891	0.1872	0.9593	0.4155	0.4079	0.9219
**20-80**	0.9080	0.2547	0.9724	0.5037	0.5815	0.9298
**30-70**	0.9076	0,3278	0.9655	0.5549	0.6037	0.9352
**40-60**	0.9224	0.3600	0.9786	0.5936	0.6274	0.9386
**50-50**	0.9223	0.3447	0.9800	0.5812	0.6330	0.9373

Supplementary Table 20: Performance of DEEP-VISTA model using 351 sequence characteristics and different portions of training and testing samples. In the first column we report % portion of training and % portion of testing samples.

Gm12878	ACC	SEN	SPE	GM	PPV	NPV
**10-90**	0.8446	0.8390	0.8451	0.8420	0.3504	0.9814
**20-80**	0.8403	0.8464	0.8397	0.8430	0.3452	0.9821
**30-70**	0.8379	0.8450	0.8372	0.8410	0.3415	0.9818
**40-60**	0.8364	0.8343	0.8366	0.8354	0.3379	0.9806
**50-50**	0.8375	0.8409	0.8372	0.8390	0.3406	0.9814

Supplementary Table 21: Promoter Overlap Fraction in actual number of bases using well-known TSS and Pol II ChIP-Seq data. In the parenthesis we report % fraction.

**Program**	**Percentage of predicted enhancer bases with TSS+Pol II regions in Hela**	**Percentage of predicted enhancer bases with TSS+Pol II regions in K562**
DEEP-ENCODE	2,305 (0.97%)	3,177 (1.12%)
CSI-ANN	4,967 (1.85%)	4,342 (1.25%)
RFECS	216 (0.02%)	334 (0.02%)
ChromHMM	78 (0.01%)	61 (0.001%)
Segway	2,040 (0.16%)	2,719 (0.09%)

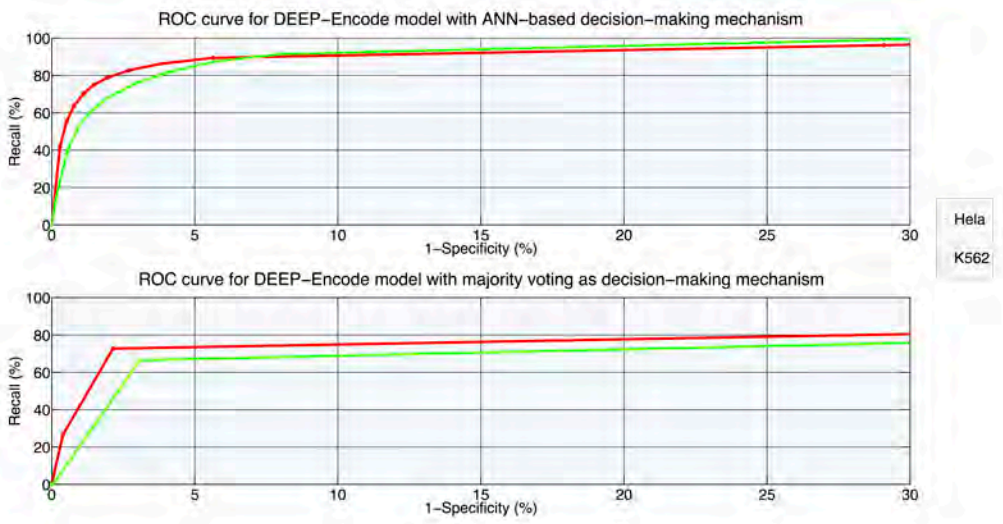

Supplementary Figure 1: ROC curve for DEEP-ENCODE model with voting in the final layer. For convenience we plot the same ROC curve using the ANN decision-making mechanism.

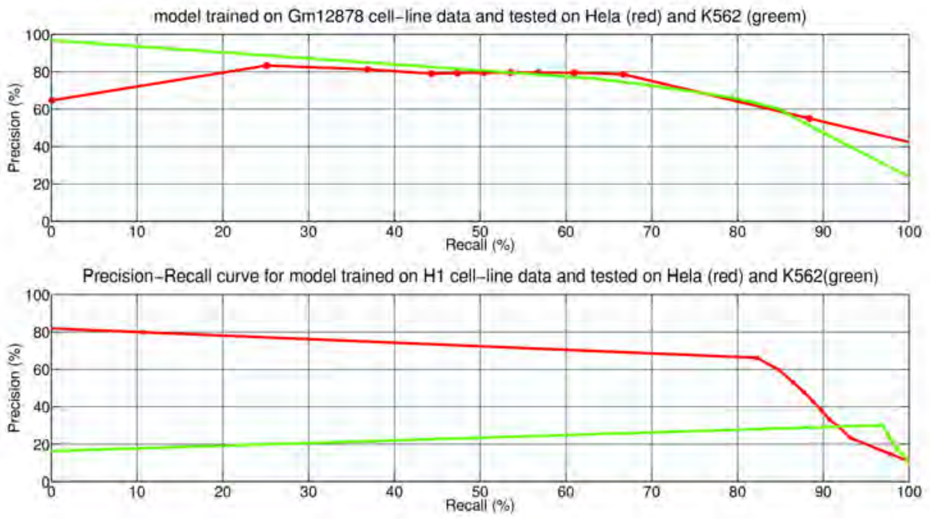

Supplementary Figure 2: Precision-Recall curves for individual cell-line models.

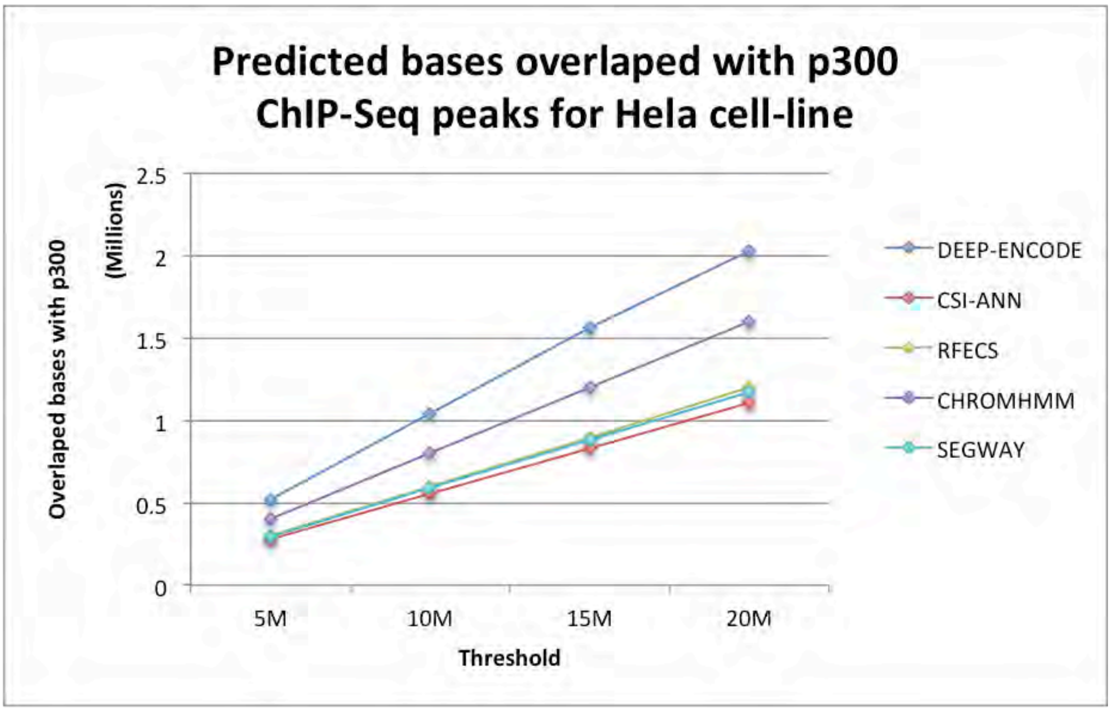

Supplementary Figure 3: Evaluating performance of the studied programs in Hela cell-line using different thresholds for the predicted bases. For all the studied programs we sample randomly 5M (M stands for millions), 10M, 15M and 20M predictions and we report overlap with p300 ChIP-Seq peaks.

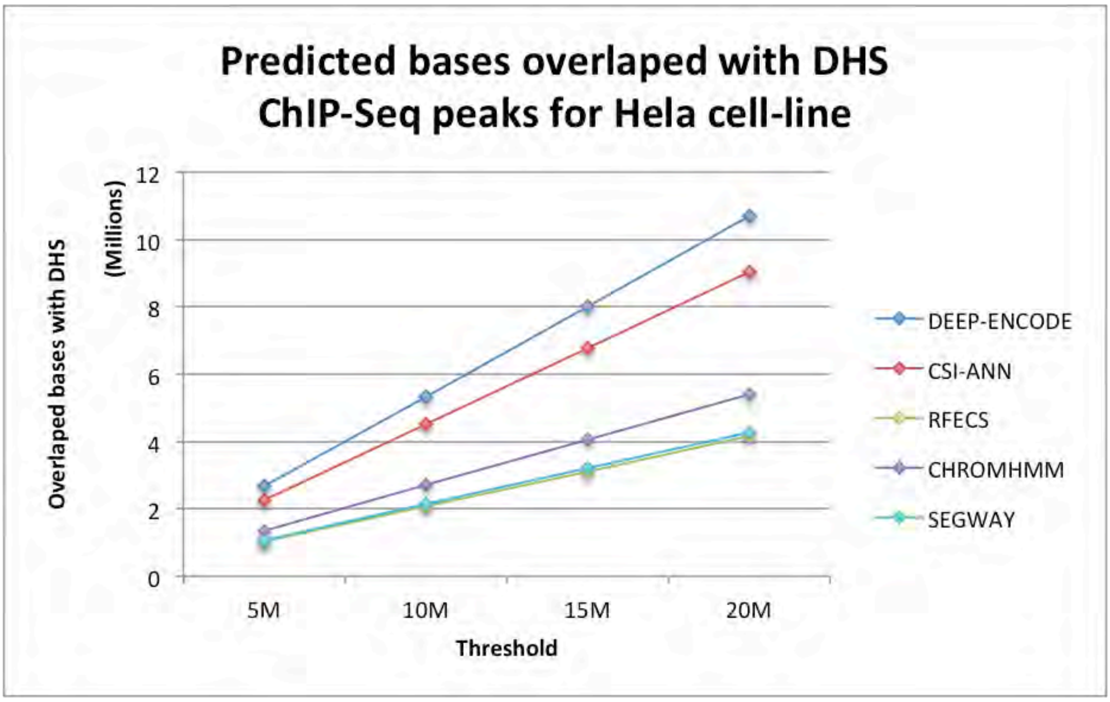

Supplementary Figure 4: Evaluating performance of the studied programs in Hela cell-line using different thresholds for the predicted bases. For all the studied programs we sample randomly 5M (M stands for millions), 10M, 15M and 20M predictions and we report overlap with DHS ChIP-Seq peaks.

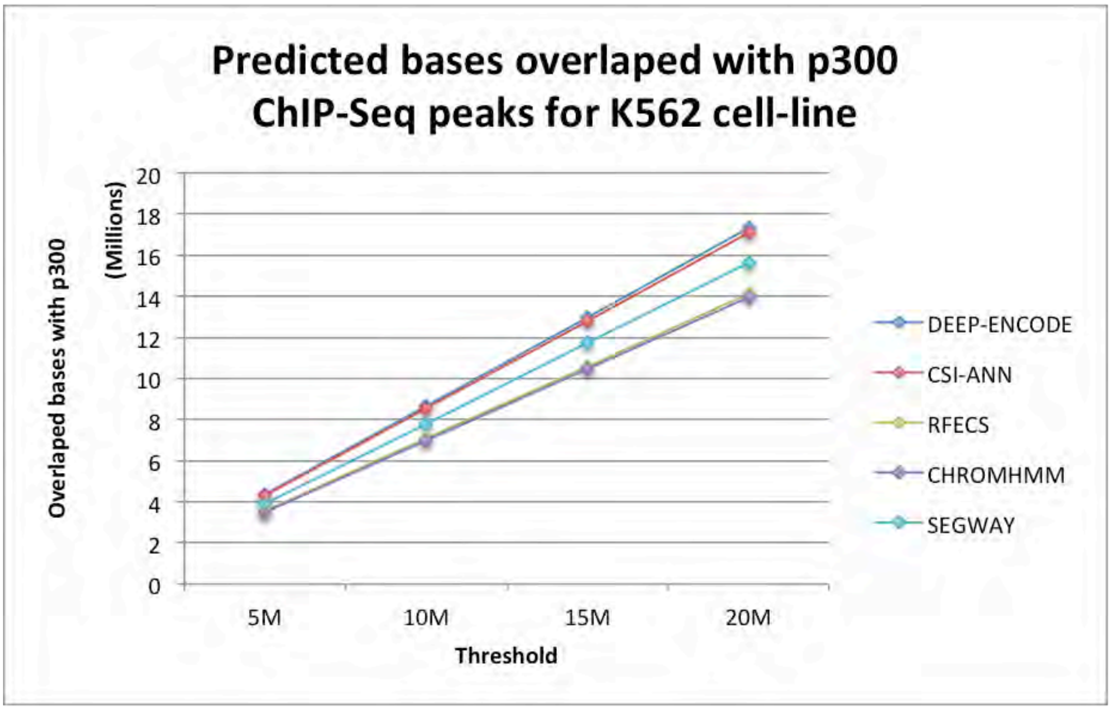

Supplementary Figure 5: Evaluating performance of the studied programs in K562 cell-line using different thresholds for the predicted bases. For all the studied programs we sample randomly 5M (M stands for millions), 10M, 15M and 20M predictions and we report overlap with p300 ChIP-Seq peaks.

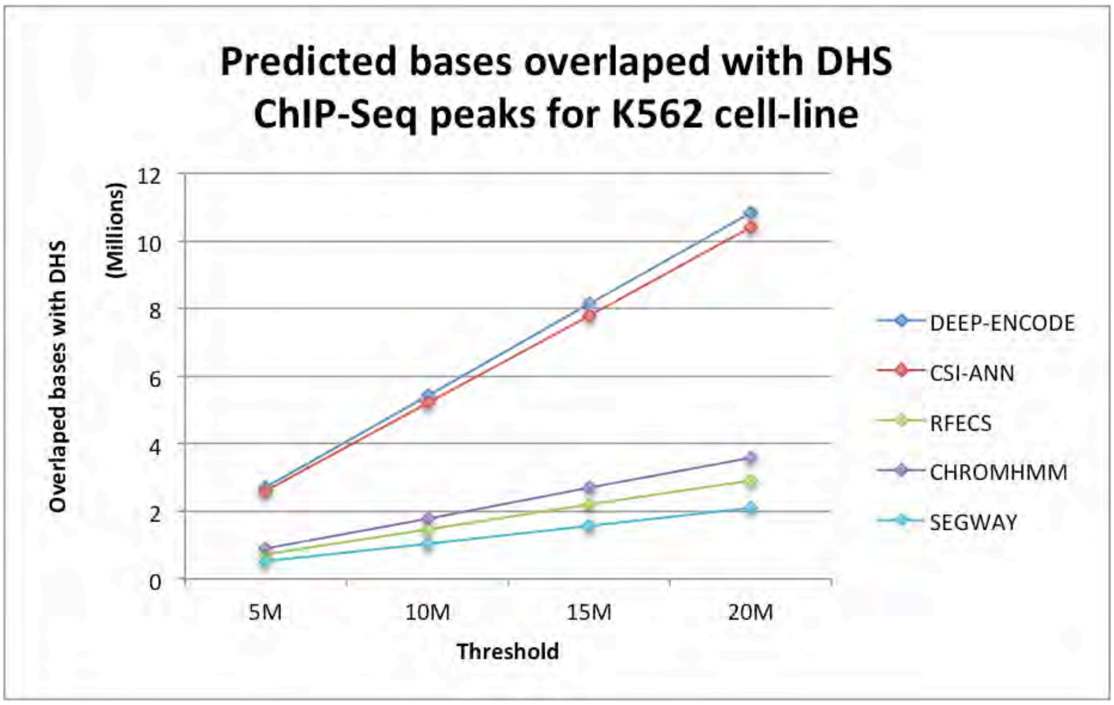

Supplementary Figure 6: Evaluating performance of the studied programs in K562 cell-line using different thresholds for the predicted bases. For all the studied programs we sample randomly 5M (M stands for millions), 10M, 15M and 20M predictions and we report overlap with DHS ChIP-Seq peaks.

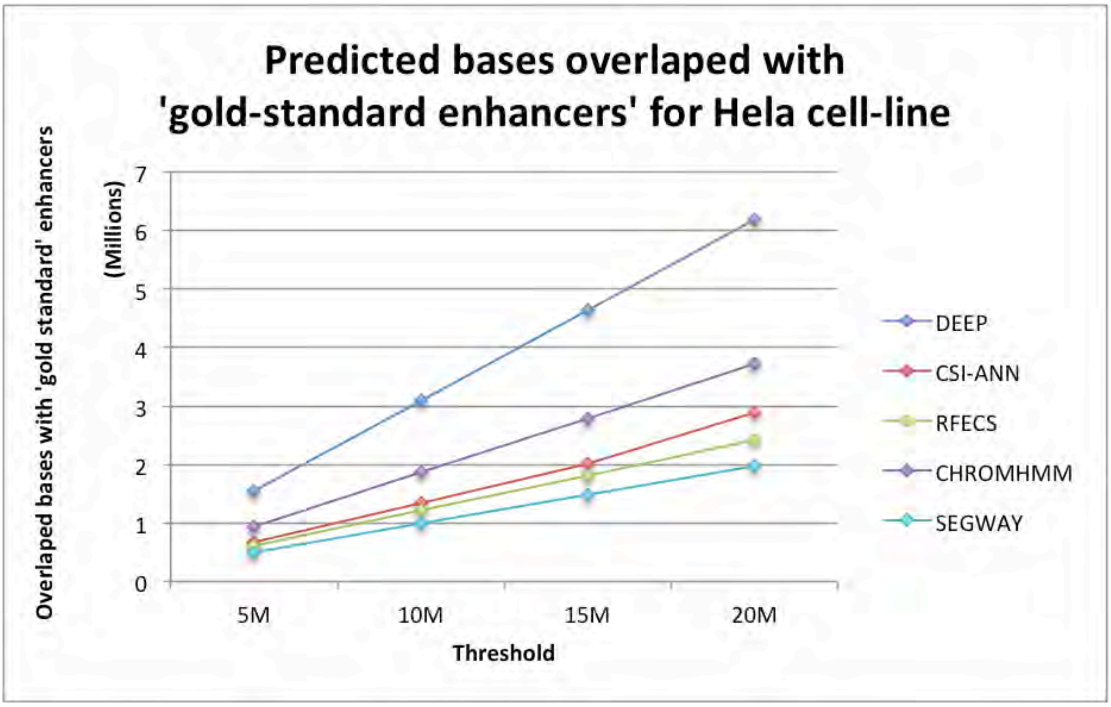

Supplementary Figure 7: Evaluating performance of the studied programs in Hela cell-line using overlap with 'gold-standard' enhancers. For all the studied programs we sample randomly 5M (M stands for millions), 10M, 15M and 20M predictions and we report overlap with genome-wide predictions.

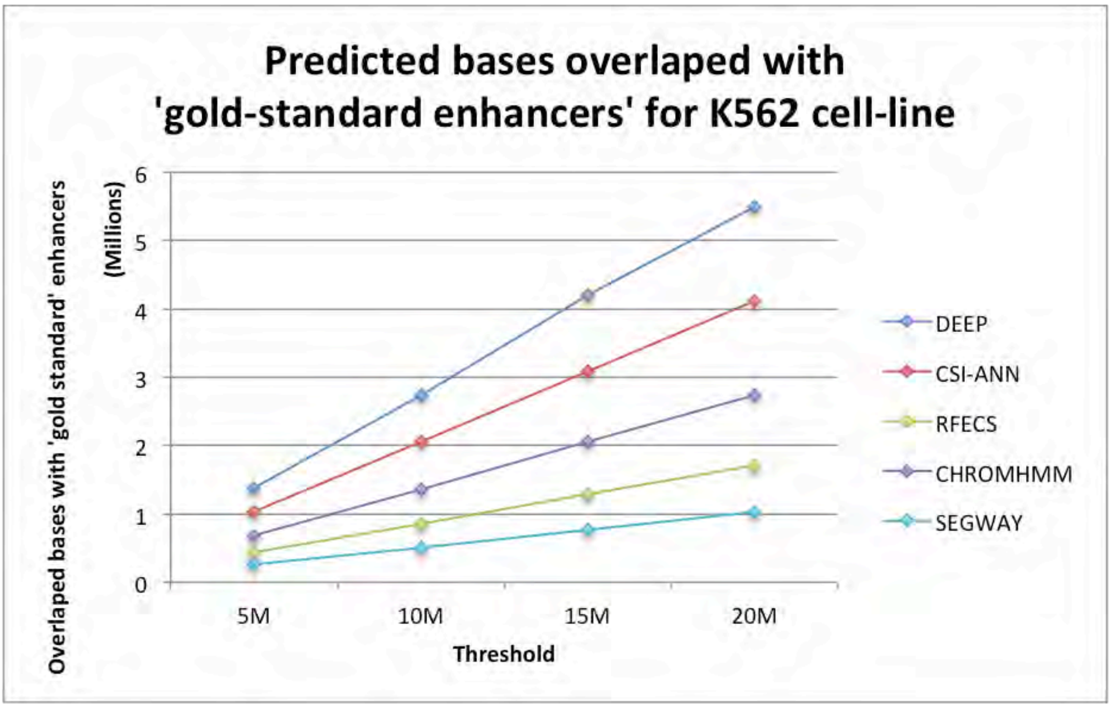

Supplementary Figure 8: Evaluating performance of the studied programs in K562 cell-line using overlap with 'gold-standard' enhancers. For all the studied programs we sample randomly 5M (M stands for millions), 10M, 15M and 20M predictions and we report overlap with genome-wide predictions.
